# Natural Volcanic Material as a Sustainable Photocatalytic Material for Pollutant Degradation under Solar Irradiation

**DOI:** 10.3390/ma15113996

**Published:** 2022-06-03

**Authors:** María Emma Borges, Silvia Navarro, Héctor de Paz Carmona, Pedro Esparza

**Affiliations:** 1 Chemical Engineering Department, University of La Laguna, Avda. Astrofísico Fco. Sánchez s/n, 38200 La Laguna, Tenerife, Spain; alu0100544756@ull.edu.es (S.N.); hpazcarm@ull.edu.es (H.d.P.C.); 2 Chemistry Department, University of La Laguna, Avda. Astrofísico Fco. Sánchez s/n, 38200 La Laguna, Tenerife, Spain; pesparza@ull.edu.es

**Keywords:** photocatalysis, volcanic materials, pollution, water cleaning

## Abstract

Recently, photocatalysis has been demonstrated as a solid approach for efficient wastewater cleaning. Using natural materials as photocatalysts means a promising solution to develop green catalysts for environmental purposes. This work aimed to study the suitability of a natural volcanic material (La Gomera, Canary Islands, Spain) as a photocatalytic material for the degradation of pollutants in wastewater with solar energy. After analysing the properties of the natural material (BET surface 0.188 m^2^/g and band-gap of 3 eV), the photocatalytic activity was evaluated at laboratory and pilot plant scale for the degradation of methylene blue (MB) in water (50 mg L^−1^), at 20 °C, during a period of 4 h, under UV/Vis light and solar irradiation. Photolytic and adsorption studies were developed to distinguish the photocatalytic contribution to the wastewater decontamination process by photocatalysis. Our results enable us to determine the viability of black sand as a photocatalytic material activated by solar irradiation (photodegradation of MB up to 100% by using solar energy), developing a natural and green photocatalytic system with significantly high potential for application in a sustainable wastewater cleaning process.

## 1. Introduction

Within the European Green Deal, water protection is one of the current priorities of the European Economic Community, which establishes a framework for the Community Action in Water policy. One of its commitments pledges to ensure the progressive reduction of groundwater pollution due to its potential use for human consumption and other services [1,2,3]. Thus, it is mandatory to develop suitable and modern methods for cleaning water, more efficient than conventional wastewater treatment techniques [4,5].

In the frame of efficient wastewater treatment techniques, the advanced oxidation processes (AOPs) have received much attention during the last few years [6,7,8,9]. This fact is particularly true for photocatalysis, demonstrated as a promising approach for wastewater cleaning due to its high efficiency and low cost [10,11]. This AOP method consists of the degradation of the pollutants through reaction with hydroxyl radicals (OH), which are generated by using solar energy [12].

**Figure 1:**
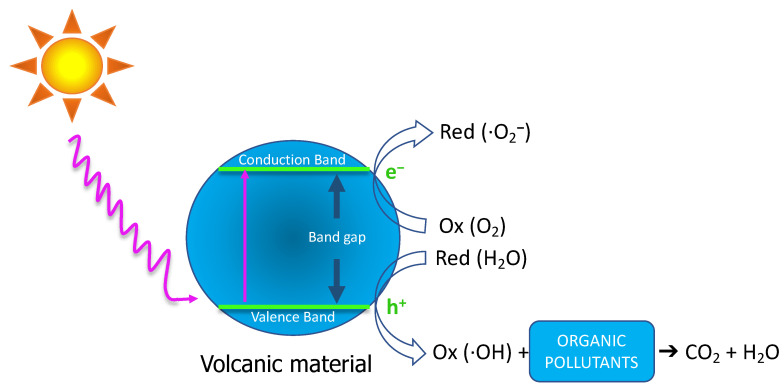
Mechanism schema of photocatalytic degradation of organic pollutants into CO_2_ and H_2_O in photocatalytic active materials.

According to this mechanism, the first step involves activating the valence band’s electrons and their migration to the semiconductor’s conduction band under a proper light source. Then, the positive holes react with water molecules to produce the hydroxyl radicals, which can degrade organic pollutants and mineralise the water due to their high reactivity. The excited electrons in the valence band are also reactive with dissolved oxygen species, generating superoxide radicals that also decompose pollutant compounds [12,13,14,15,16]. Analogous to biofuel production [17], the current research on photocatalysis focuses on developing new materials with high activity and stability. Figure 1 shows a simplified diagram of the photocatalytic mechanisms of pollutant degradation.

Previous studies in this area of research have reported that metal-transition oxides such as TiO_2_, WO_3_ or V_2_O_5_ (also known as semiconductors materials) are potential photocatalysts [18,19,20]. TiO_2_ is probably one of the most studied materials in the photocatalysis field due to the high potential for pollutant degradation, solid chemical stability and non-toxic properties [21,22]. In this sense, researchers have already reported the use of supported and unsupported TiO_2_ catalysts for the photodegradation of numerous contaminants, such as Toluene, m-Xylene and n-Butyl Acetate [23,24,25,26], and its disinfection properties towards bacteria by using suspended and immobilised silver–TiO_2_ or carbon nanomaterial–TiO_2_ [27,28].

Recent studies in this area of research have pointed to natural and waste materials as efficient materials in advanced photocatalysis systems. This fact is based on several factors, such as their unique porosity structure, lightweight property and safety of manipulation [29,30]. However, only a few studies have focused on using natural or waste materials in wastewater treatment [31,32,33], indicating a knowledge gap. The use of natural materials for catalysis purposes represents an exciting approach to EU commitments, in the framework of the Green Deal and the circular economy action plan (CEAP), current priorities of the EU. This lack of information makes the suitability study of natural materials mandatory for environmental and energy purposes.

In this way, this work builds on our previous research [24,25,31,32,33] by studying black volcanic sand as a suitable material for pollutant photodegradation in a pilot plant-scale photoreactor (artificial UV/visible light and natural sunlight). The suitability study was performed based on the material’s properties and activity for pollutant adsorption and photocatalysis. Our results enable us to describe the viability of using this natural material as a photocatalyst for wastewater treatment, compared to other metal-oxide catalysts, amply described in the references.

## 2. Materials and Methods

### 2.1. Photocatalyst Characterisation

Naturally occurring volcanic material, black volcanic sand, with 0.200–0.400 mm particle size, was characterised using several methods to evaluate its photoactivity under UV/vis and solar irradiation. The volcanic material was collected in La Gomera (Canary Islands, Spain) during the summer season. Local temperature is always in the same range (15–25 °C) for almost the whole year.

X-ray diffraction (XRD) was used to identify the composition of the phases (X’Pert Pro analytical; Cu K α radiation and λ = 1.2 Å). The textural properties were characterised by Hg porosimetry using an AutoPore IV mercury porosimeter (Micromeritics, Norcross, GA, USA) and N_2_ physisorption using a Gemini V (Micromeritics, Norcross, GA, USA). The specific surface area was calculated from the nitrogen adsorption–desorption isotherms. The potential as a photocatalyst was evaluated by UV/vis spectra (Varian Cary 3 UV/vis spectrometer with integration sphere, Agilent Technologies, Santa Clara, CA, USA). The spectra were recorded in diffuse reflectance mode and transformed to equivalent absorption (Kubelka–Munk units). Finally, the pollutant degradation was verified by FT-IR spectroscopy (AVATAR 360 FR-IR).

### 2.2. Experimental Setup and Photocatalytic Tests

The experimental setup was similar to the authors’ previous works [24,25]. The following includes a brief description of the used photocatalytic reactors, irradiation lamps and operating conditions.

**Figure 2:**
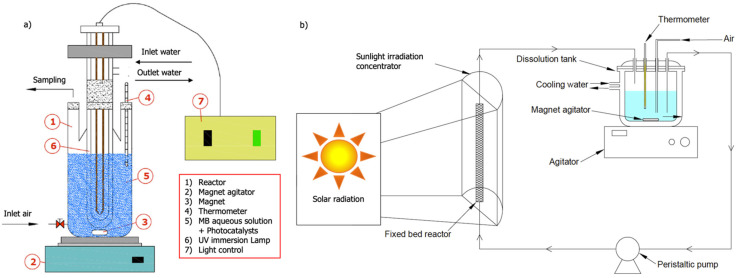
Schema of photoreactors. (**a**) Batch photoreactor; (**b**) fixed bed photoreactor.

The photocatalytic experiments were performed using artificial UV/visible light and natural sunlight in a laboratory and pilot plant scale. For laboratory-scale experiments, the reaction system was composed of a 250 mL stirred photoreactor with UV and simulated solar radiation using a Hg lamp (Heraeus TQ-150, 150 W—no optical filter, Hanau, Germany) and Xe lamp (Hamamatsu L2274, 150 W—no optical filter, Ammersee, Germany) or a parabolic sunlight irradiation concentrator for collecting and diffusing solar radiation, respectively. Figure 2 shows a schema of the batch and fixed bed photoreactors.

Another part of the experimentation was carried out using solar radiation, so the experimental facility was moved outside, replacing the radiation-emitting lamp with solar radiation. The experiments developed under these conditions (solar photocatalysis experiments, EFS) were performed with a tank-type stirred reactor made of quartz to avoid the filtering of specific wavelengths coming from solar radiation, containing 50 mL of pollutant solution and the photocatalytic material under study, maintaining the conditions of continuous agitation and air bubbling. As a difference, in these photocatalysis experiments, the reactor was equipped with a parabolic solar collector whose goal was to concentrate the incident solar radiation.

The photocatalytic activity of the studied material for pollutant degradation was measured using methylene blue (MB: 50 mg L^−1^) as a model molecule of the organic contaminant in water and 3 g L^−1^ of tested material as a photocatalyst. The photocatalyst dosage was set based on the optimum dosage for the catalyst particle size in order to use the maximum amount of catalyst and minimise the shadow effect of catalyst particles. This value was obtained as optimal for wastewater pollutant degradation purposes [32]. The photocatalytic material was placed inside the reactor, suspended in the aqueous solution with no appreciable floatability effect. The same operating conditions were set for all tests developed: pH 5–6, 20 °C, irradiation time of 4 h and continuously pumping air into the photoreactor to provide oxygen. Several samples were obtained from the reactor as the irradiation time progressed, and they were analysed, after centrifugation to separate the photocatalyst, by spectrophotometry to study the evolution of the concentration of the contaminant in wastewater during the process of photocatalytic degradation.

Pollutant degradation was evaluated by sampling the wastewater during testing, centrifuging to remove the photocatalyst and determining the MB concentration with a UV/vis spectrophotometer (Varian model Cary 50; λ = 663 nm). Experiments with sunlight were performed similarly to the previous experiments of the authors [25,32,33]. The experiments with sunlight were carried out in the stirred tank-type quartz photoreactor. A parabolic collector was used to concentrate the radiation that reached the photocatalyst since its incidence improved the photocatalytic efficiency. An incident radiation analysis was then performed, and the temperature in the photoreactor was recorded.

**Figure 3:**
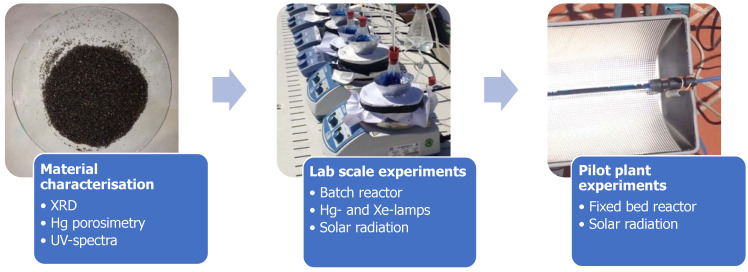
Flow chart of the experimental procedure.

When the photocatalysis experiments under solar irradiation were carried out in a packed bed reactor, the type of stirred reactor was modified, using a continuous photoreactor with recirculation that allowed us to optimize the incidence of solar radiation. This type of photoreactor consisted of an elongated, cylindrical tube made of glass. The particles of the photocatalytic material under study were packed, thus constituting the packed bed reactor system. Figure 3 shows a flow chart of the experimental procedure.

## 3. Results and Discussion

### 3.1. Catalyst Characterisation

**Figure 4:**
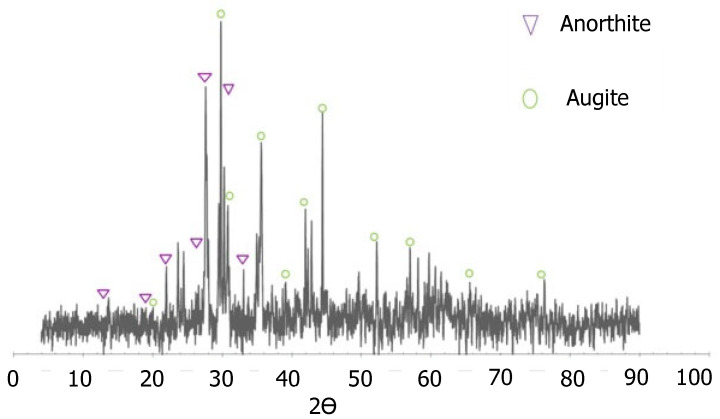
XRD diffraction pattern of volcanic material (black sand).

XRD analysis was used to identify the main phases of the natural material used as a photocatalyst. Figure 4 shows the XRD diffraction patterns. Detected main phases are marked in the figure, i.e., augite (Ca (Mg, Fe) Si_2_O_6_) and anorthite ((Ca, Na) (Al, Si) 2Si_2_O_8_) in a composition of 28 and 69%, respectively. The other phases were identified as amorphous silica (broad peaks between 25° and 35°).

**Figure 5:**
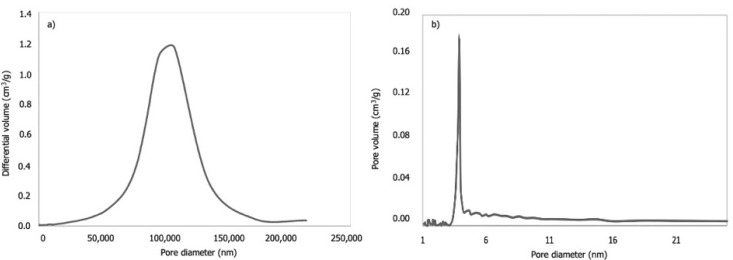
Pore size distribution of volcanic material (black sand), (**a**) determined by Hg porosimetry and (**b**) determined by N_2_ physisorption.

Hg porosimetry and N_2_ physisorption were used to analyse the photocatalytic material’s pore volume distribution and specific surface, respectively. Figure 5 shows the pore size distribution of black sand by Hg porosimetry and N_2_ physisorption. The black sand shows a predominance of macropores in the structure of the material. Adsorption isotherms of N_2_ result in a specific surface of 0.188 m^2^/g and mean micropore diameter of 3.9 nm. Meso- and macroporous structure analysis is shown in Table 1, including the porosity and density of the studied natural material.

**Figure 6:**
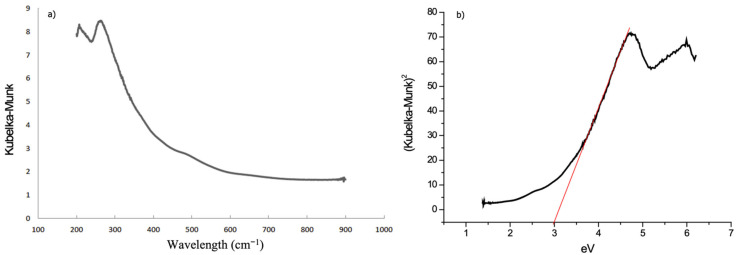
UV/vis spectra in Kubelka–Munk units vs. wavelength (**a**,**b**) photonic energy (eV). Red line corresponds to band-gap determination.

Before performing the experiments, the potential of the volcanic material as a photocatalyst was first evaluated using a UV/vis spectrometer to determine the band-gap. Figure 6 shows the obtained spectra expressed in Kubelka–Munk units (equivalent units of light absorption) vs. wavelength (cm^−1^) and photonic energy (eV). The red line shows the determination of the material’s band-gap.

As Figure 6 shows, the volcanic material has a band-gap that corresponds to an energy of 3 eV, which can be activated with UV radiation at 450 nm. Comparing these results with references (Table 2), the band-gap of the volcanic sand is significantly lower than that of TiO_2_. Together with an increase in the activation wavelength values, this fact demonstrates the potential photocatalytic activity of the material. Thus, it is possible to claim the viability of using this material for photocatalysis purposes with sunlight as an energy source. This fact also means a more sustainable application with significant energy savings. The 44% of the total energy emitted by the Sun corresponds to the visible range of wavelengths.

### 3.2. Photocatalytic Activity

The type of photocatalytic material has a key influence on the photocatalytic degradation of the treated pollutant. Therefore, in this research, studies of the direct photolysis of the contaminant, adsorption of the contaminant on the surface of the photocatalytic material and photocatalytic degradation of the contaminant were performed in order to compare the results obtained and to check whether the photocatalytic process efficiently degrades the contaminant in the water.

It is important to consider the advantage that the photocatalytic process brings to wastewater decontamination since it allows the contaminant to be eliminated from the wastewater definitively, completely mineralising the contaminant. In contrast, the adsorption process is only capable of removing it from the effluent to be treated. That is, it separates it from the wastewater, but the pollutant molecule is still present on the surface of the adsorbent material. Evaluating the direct photolysis of the contaminant is also necessary to know the exclusive effect that irradiation produces on the contaminant.

Thus, considering photocatalysis as the preferred method to mineralise the pollutant in wastewater, a blank run was determined by the direct photolysis of the pollutant in the absence of the catalyst. Catalyst stability has been evaluated according to the number of successive reuse cycles that significantly affect the catalyst’s activity.

#### 3.2.1. Degradation of Pollutants by Photolysis

**Figure 7:**
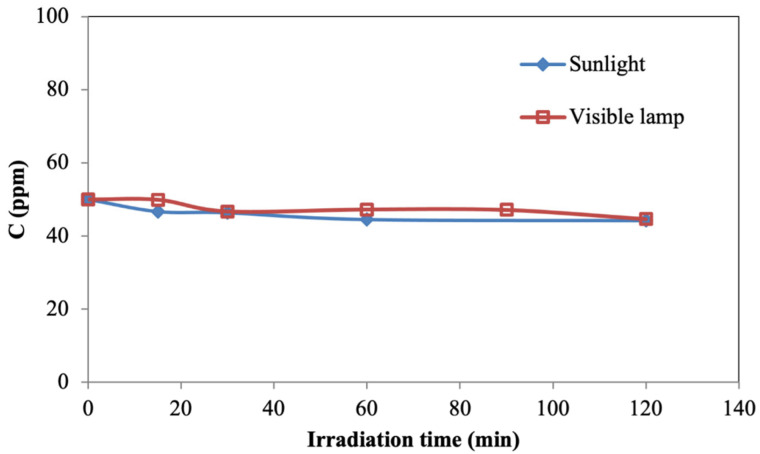
Pollutant degradation by photolysis in absence of photocatalyst, using both sunlight and visible lamp.

Prior to the photocatalytic experiments, the degradation of the pollutant by photolysis in the absence of the catalyst (i.e., blank runs) was evaluated. For this purpose, the polluted water sample was irradiated using UV/visible wavelength range light in a stirred photoreactor with a radiation-emitting lamp or under direct solar radiation. Figure 7 shows the contaminant concentration in water varying with irradiation time using both sunlight and artificial light.

As mentioned in references [34,35,36], the radiation emitted by the visible lamp was not sufficient to achieve the significant degradation of the pollutant. Similar behaviour was observed when using sunlight, with negligible degradation. Thus, it is possible to claim that photolysis was always low and insignificant in pollutant degradation without a photocatalytic material.

#### 3.2.2. Adsorption Properties

The absorption study is of vital importance to understand to what extent the photocatalyst is able to eliminate the contaminant since, through the exclusive occurrence of the adsorption phenomenon to be studied below, the contaminant would not be degraded or disappear from the effluent. Rather, it would remain attached to the surface of the photocatalytic material as a consequence of its adsorbent capacity, taking into account the interactions between the contaminant molecules and the surface of the photocatalytic material studied [37].

**Figure 8:**
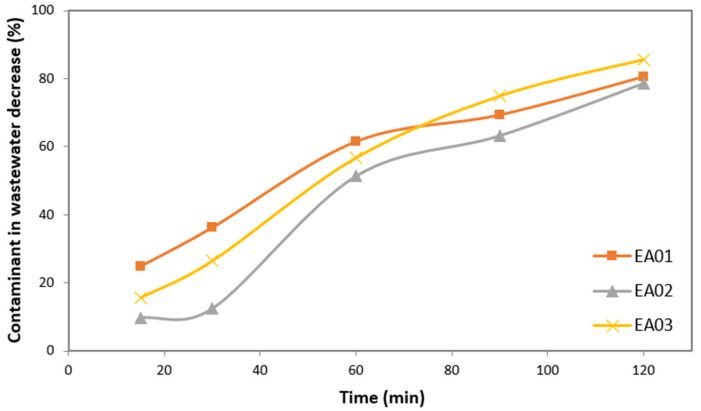
Pollutant decreases by adsorption of the volcanic material (black sand) in absence of light (EA01: pH 5.36, photocatalyst 0.12 g/L, MB 50 ppm; EA02: pH 5.44, photocatalyst 0.12 g/L, MB 50 ppm; EA03: pH 5.36, photocatalyst 0.12 g/L, MB 50 ppm).

Thus, after the blank run, the next step developed was the study of pollutant adsorption on the catalytic material without a light source. This point is necessary to distinguish between the pollutant decrease due to adsorption and photodegradation. The pollutant molecules do not disappear but remain adsorbed to the material surface [38]. This series of experiments was carried out under the same conditions described in the Materials and Methods section. Figure 8 shows the pollutant decrease in wastewater by adsorption onto the photocatalytic material studied.

Since sand is a good adsorbent material, studies carried out in the absence of light (adsorption) reflect considerable percentages of adsorption of the contaminant on the surface of the photocatalytic material. Thus, it can be seen from Figure 8 that the pollutant decrease by adsorption was up to 80% at the end of the experiments and the contaminant remained attached to the surface of the material adsorbent.

These results make sense considering the nature of this material, i.e., sand is a good natural adsorbent material. This phenomenon has been reported in references [37,39], pointing to the importance of this factor to the photocatalytic pollutant degradation rate. Due to this fact, volcanic sand was analysed by FT-IR after the photocatalytic experiments to distinguish between adsorption and photocatalysis activity.

#### 3.2.3. Photocatalytic Properties

**Figure 9:**
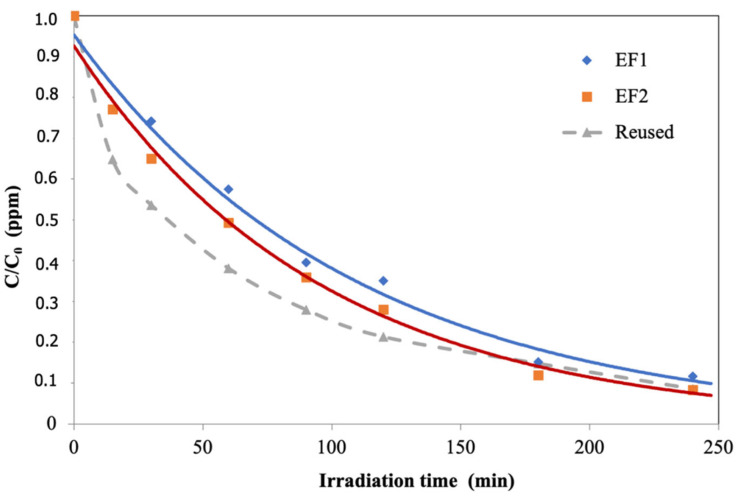
Contaminant in wastewater degradation by photocatalysis using black sand as photocatalyst under visible light. Experiments developed in a narrow pH range (EF1—5.80, EF2—5.84) and with reutilization of the photocatalytic material in several runs.

As described in the Materials and Methods section, the photocatalytic properties of the studied natural material were measured using artificial light and sunlight. Figure 9 shows the pollutant degradation during irradiation, expressed as the C/C_0_ ratio. These results show that the black volcanic sand catalyst was photochemically active for pollutant degradation (i.e., photodegradation), with values up to 100% after 4 h of testing. Several experiments performed always showed similar values, even in the case of photocatalyst reuse, obtaining the total photodegradation of the pollutant after 4 h. Thus, it is possible to claim the reusability of volcanic sand for a continuous-use regime under visible light.

As described in the Section 2, the pH range was 5–6. This range was chosen due to previous research in the literature showing that pH can influence the percentage of degradation [40], and a slightly acidic pH enhances the photocatalytic degradation of MB as a water pollutant [41]. Thus, it was possible to assume that photocatalysis is more efficient in an acidic medium (pH between 3 and 5) and that the variation in pH affects the surface properties of the catalyst. This phenomenon was manifested in the displayed results, whose pH value was above these optimal values (experiments developed around values between 5 and 6 pH units). The rate of degradation was always significant, producing a variation in the degradation rate of the contaminant between experiments.

After verifying the high potential of the natural material as a photocatalyst, experiments were carried out to evaluate the capacity of the material when used in direct sunlight, which would constitute a more sustainable process for wastewater decontamination.

**Figure 10:**
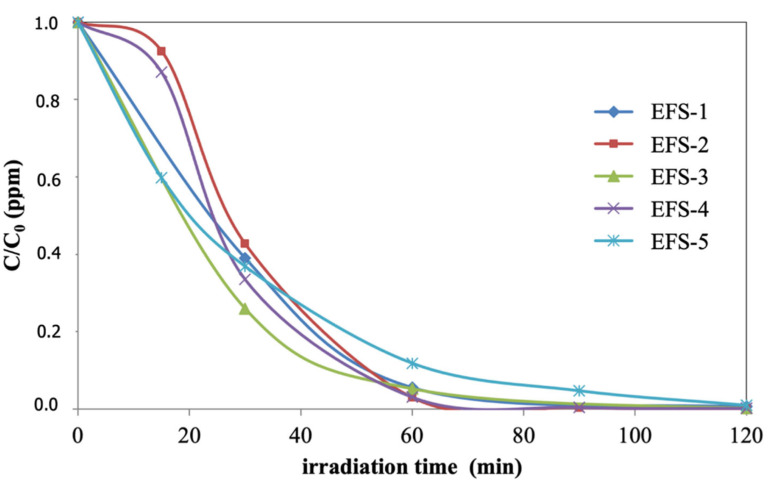
Wastewater pollutant degradation by photocatalysis using black sand as a photocatalyst and sunlight activating the photocatalyst (expressed in C/C_0_ units) for several experiments under varying solar irradiation (experimental conditions in Table 3).

In the case of using sunlight, the photocatalytic material also showed the significant photodegradation of pollutants. Figure 10 shows the pollutant degradation in wastewater during irradiation, expressed as the C/C_0_ ratio. Indeed, sunlight instead of artificial visible light significantly increased the photocatalytic activity, obtaining up to 100% of pollutant photodegradation after 60 min of testing. Moreover, there seems to be a dependence between the photodegradation rate and the zenith angle (Table 3). When performing these experiments under sunlight, it was possible to monitor how the pollutant photodegradation rates were related to lower global radiation intensities and zenith angles.

**Figure 11:**
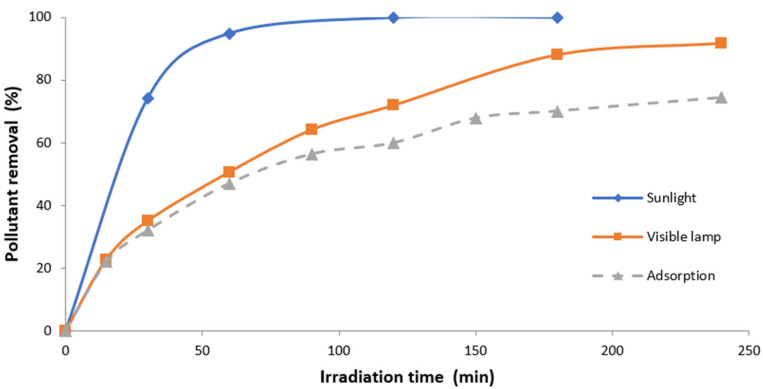
Pollutant in wastewater removal/degradation by adsorption/photocatalysis.

The activity of the catalytic material for pollutant degradation by photocatalysis (using artificial and sunlight) was compared to the adsorption capacity of the studied material as a photocatalyst. Figure 11 shows the pollutant degradation and pollutant removal in wastewater during photocatalysis and adsorption experiments, respectively. From the results shown in this figure, it can be seen that the volcanic material showed significant photocatalyst activity under sunlight (100% after 120 min) and simulated solar radiation (90% after 180 min). These values were consistently higher than the pollutant removal by adsorption on the material surface (60% after 180 min).

**Figure 12:**
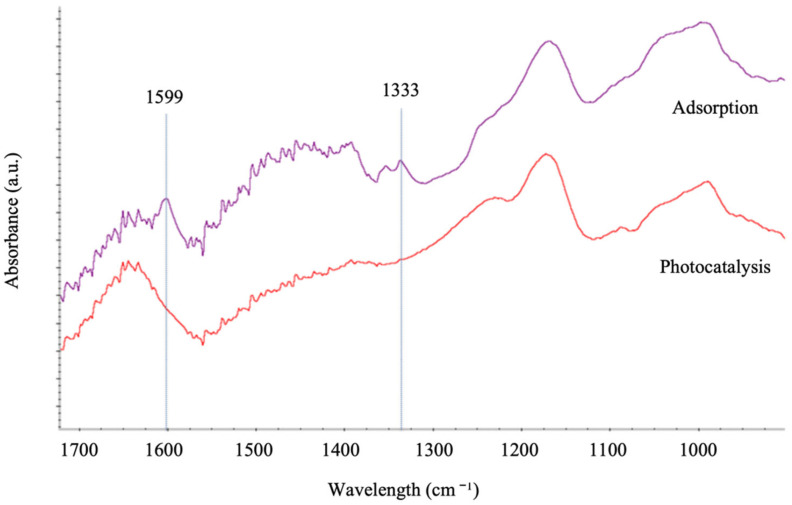
FT-IR spectra of volcanic material (black sand) after adsorption and photocatalytic experiments.

The photocatalytic degradation of the contaminant due to the activation of the photocatalytic material by the input of light can be distinguished from the adsorption phenomenon by analysing the presence of the contaminant (MB) on the catalytic surface. Figure 12 shows the FT-IR spectra of the black volcanic sand used for the pollutant adsorption experiments and the materials used as photocatalysts for the pollutant degradation experiments. As seen in the spectra obtained for both materials, the spectrum of the material used in the adsorption experiments (without light) showed characteristic peaks corresponding to the methylene blue molecule adsorbed on its surface. When the same material was used in the photocatalysis experiments (in the presence of light), these peaks did not appear in the spectrum, indicating that the degradation of the contaminant occurred. The absence of MB characteristic peaks (1599 and 1333 cm^−1^, which correspond to CAr-N and C= bonds, respectively) in the photocatalyst samples used in the photoreactor indicates the complete photodegradation of the pollutants [32,33].

Therefore, it was possible to verify that sand is a good adsorbent material. However, in the presence of a light source, it can act as a photocatalyst for the total degradation of contaminants in water. The natural material under study in this work was shown to have optimal photocatalytic activity, similar to semiconductor materials with photocatalytic activity recognised at an industrial level, such as titanium oxide.

**Figure 13:**
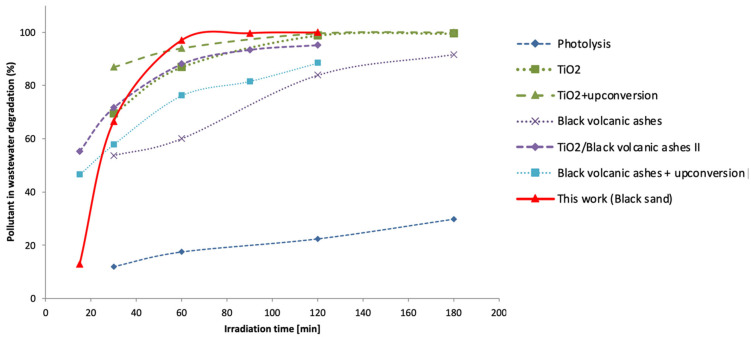
Photoactivity of the material studied under sunlight compared to other natural materials studied in the literature; up-conversion systems were studied in the literature to improve the photoactivity of semiconductor materials in the wavelength range of the Sun.

It also presented improved photoactivity under sunlight compared to other natural materials studied in the literature [31,33], even when the photocatalytic activity of those materials was enhanced by using light up-conversion systems for better use of sunlight [32] (Figure 13).

**Figure 14:**
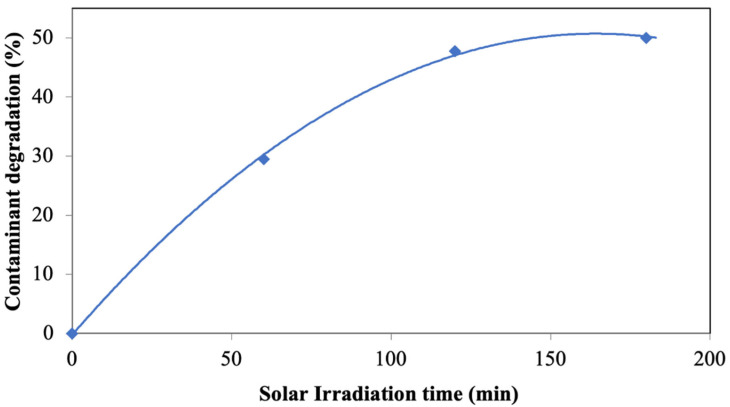
Wastewater decontamination by photocatalysis in photoreactor system using solar irradiation with packed bed configuration for photocatalyst.

In concordance with the results obtained at the laboratory scale, photodegradation of pollutants was also carried out using a pilot plant-scale photoreactor with photocatalyst particles configured in a packed bed. Figure 14 shows the obtained results, observing up to 48% of pollutant photodegradation after 120 min of irradiation. These results indicate excellent photocatalytic activity at the pilot plant scale and under a continuous regime.

At this pilot plant scale, it is intended to achieve higher contact of the photocatalyst with the pollutant to be degraded while achieving a greater exposure surface of the photocatalytic material to the incident radiation. This reaction system achieves more scalable results from an industrial point of view since having the photocatalyst in a packed bed improves the efficiency of the process compared to the laboratory scale. It also allows the minimisation of the deterioration of the photocatalytic particles and allows continuous solar photocatalytic treatment. Therefore, the application of this methodology seeks to obtain a more economical and sustainable treatment of wastewater by using solar radiation.

## 4. Conclusions

This work studied the suitability of a natural material (black sand of volcanic origin) as a potential photocatalyst to degrade pollutants in wastewater sustainably. The catalyst was tested in laboratory- and pilot plant-scale photoreactors using artificial UV/visible light and natural sunlight, with a low irradiation time and temperature. The photocatalyst activity was suggested by proper material characterisation and confirmed by significant values of pollutant photodegradation. In this sense, the photodegradation rates were up to 80 and 100% after 60 min of irradiation when using stirred photoreactors with artificial and sunlight, respectively. Moreover, the pilot plant-scale photoreactor system showed significant values of photodegradation in a continuous regime, with advantages from an industrial point of view, with the capability of treating high flows of wastewater in continuous operation. Obtained results describe the viability of using natural materials as photocatalysts for wastewater treatment, with good efficiency for pilot plant-scale photoreactors using sunlight as a sustainable decontamination process. This work also points to this natural material, and the photoreactor configuration studied, as more efficient wastewater treatment systems than the current technologies.

## Figures and Tables

**Table 1:** Characterisation of volcanic material (black sand).

Parameter	Value
*N*_2_* physisorption*	
BET specific area, m^2^/g	0.188
*Hg porosimetry*	
Median pore diameter (Area), nm	73.4
Average pore diameter (4V/A), nm	5852.4
Density_bulk_, g/mL	1.6296
Density_apparent_, g/mL	2.9586
Porosity, %	44.9210

**Table 2:** Band-gap (eV) and wavelength (nm) of light activation for several photocatalytic materials.

Photocatalytic Material	Band-Gap (E_g_), eV	Wavelength, nm
TiO_2_ P25	3.2	365
TiO_2_ Merck	3.3	370
Black sand	3.0	450
Black volcanic ashes [31]	2.4	410
TiO_2_-supported black volcanic ash I [32]	3.1	385
TiO_2_-supported black volcanic ash II [32]	2.8	380
TiO_2_-supported black volcanic ash III [32]	2.9	385

**Table 3:** Operating conditions of photocatalytic experiments using black sand as photocatalyst and solar radiation as light source.

Exp.	pH	Local Time	Zenith Angle, °	Solar Radiation, KWh/m^2^
EFS-1	5.35	11:40 a.m.	43–35	1.71
EFS-2	5.01	11:40 a.m.	43–35	1.73
EFS-3	6.00	09:20 a.m.	66–44	1.17
EFS-4	6.00	11:35 a.m.	37–25	1.94
EFS-5	5.87	11:25 a.m.	37–13	1.77

## Data Availability

Not applicable.
